# Hybrid Feel-Own-Move®: protocol for an effectiveness-implementation study of a psychomotor intervention for survivors of domestic violence

**DOI:** 10.3389/fpubh.2025.1551809

**Published:** 2025-02-17

**Authors:** Joana Machorrinho, Guida Veiga, José Marmeleira, Mia Scheffers, Graça Duarte Santos

**Affiliations:** ^1^Comprehensive Health Research Center, Universidade de Évora, Évora, Portugal; ^2^Departamento de Desporto e Saúde, Escola de Saúde e Desenvolvimento Humano, Universidade de Évora, Évora, Portugal; ^3^School of Human Movement and Education, Windesheim University of Applied Sciences, Zwolle, Netherlands

**Keywords:** body–mind intervention, therapy, intimate-partner violence, women, children, hybrid, healthcare, shelter homes

## Abstract

**Background:**

Domestic violence is a public health concern, impacting the health and well-being of women and children globally. Shelter homes are one of the support services for victims’ recovery, although providing holistic healthcare in this setting remains a struggle. Feel-Own-Move® (FOM) is an evidence-based psychomotor intervention designed to help women who have experienced domestic violence reconnect with their bodies. Hybrid FOM (H-FOM) is a version of FOM that combines in-person with online sessions for both women and children living in shelter homes. To examine the effectiveness and implementation success of H-FOM are the aims of this study.

**Methods:**

This protocol details an effectiveness-implementation type I hybrid study, to be carried out in shelter homes across three European countries. Health outcomes of the participants, and the implementation success within professionals from the shelter homes and the psychomotor therapists responsible for implementing H-FOM will be assessed. Results will be analyzed through a mixed methods approach, following the conceptual model of implementation science and the RE-AIM framework.

**Discussion:**

This effectiveness-implementation study is expected to contribute to understanding H-FOM health-related effects on women and children survivors of violence, as well as to its sustainable implementation, up-scaling and integration into trauma support services and associated healthcare policy. H-FOM is expected to (i) improve the known effects of FOM on women survivors of DV, while ensuring continuity of the therapeutic process following relocation, and promoting the health and well-being of children living in the shelter homes.

## 1 Introduction

In Europe, about 19% of women have experienced domestic violence (DV) in the form of physical and/or sexual abuse by a partner, a relative or family member, with varying report rates across countries [e.g., Portugal (11.5%), Spain (15.9%) and the Netherlands (19.9%)] (1). Since DV refers to any act of physical, psychological, sexual or economic violence within an intimate relationship or family system, children living in violent family contexts are also victims, either by witnessing violent behaviors, by relating with adults with disruptive behavioral and psychological patterns, or by suffering direct abuse (e.g., humiliating physical punishments or psychological coercion) (2, 3).

Victims of DV suffer negative repercussions on their physical and mental health, identity structure and social integration (3–5). Specifically, women victims report high rates of anxiety, post-traumatic stress disorder, depression, somatic symptoms, traumatic brain injury and physical impairments (6, 7), which carry significant social and public health costs. Additionally, women face structural societal inequalities, such as lower socioeconomic status, reduced access to education, limited employment opportunities, and restrictive gender expectations (8). These factors, through social and emotional mechanisms, perpetuate the risk of domestic violence, hindering victim’s chances of recovering health and quality of life (4, 8, 9).

In parallel, children victims of DV show higher prevalence of brain damage and injuries (10, 11), physical health complaints such as somatization, eating, sleeping and pain problems (12), and emotional and behavioral problems (13), and end up with a heightened risk of developmental delay (10, 14). Research discusses the detrimental effects of DV on children and adolescents as a developmental cascade, where even short-term effects can extend and provoke long-lasting impacts in various domains, such as physical health, learning, and social–emotional development (15). Moreover, the trans-generational transmission of violence keeps feeding the cycle of violence, leading to re-victimization or violence perpetration in adulthood (16). Research suggests bodily dissociation as a negative effect of adverse childhood experiences and a mediator mechanism between those and DV victimization in adulthood (17).

DV perpetrators often deprive victims of appropriate and timely health care, of emotional and economic independence, healthy social relationships, and leisure opportunities (9). These characteristics, combined with victims’ chronic feelings of being endangered, undermine women’s and children’s possibilities and motivation to autonomously engage in health-enhancing practices (such as physical activity and self-care), leading to physical and mental health risks added to social isolation. Structural interventions that improve women’s economic well-being, relationship quality, empowerment, or social group membership, as well as the social, relational and physical protection of children are necessary to prevent and diminish DV (18).

One of the globally recognized actions to support the immediate safety and extended recovery of DV women survivors and their children is shelter homes. Shelter homes are part of victims’ support policies, offering them an opportunity to relocate, a safe place to live, with food, social counselling, legal support, employment support and in some cases psychoeducation, in addition to facilitated school process for the children. Due to shelters being a favorable context for safe trauma recovery, efforts have been made to give the residents psychological and health care. However, research suggests that women living in shelter homes still have poor general health, including trauma-related symptomatology, somatic symptoms, sedentarism, and a strong disconnection from the body, which undermine their quality of life, identity structure and decision-making processes, crucial for preventing revictimization (5, 19, 20). Advances in trauma care and related interventions suggest that the support to victims of violence must consider a more holistic approach to their health, including physical activity, body awareness, and relaxation (4, 21–23). To address this recommendation, various body–mind approaches for trauma recovery have been developed and implemented (4, 19, 23, 24). Feel-Own-Move is one of them, a psychomotor therapy approach to trauma and violence victimization.

### 1.1 Feel-Own-Move®

Feel-Own-Move® (FOM) is an innovative evidence-based approach, designed to enhance the health and well-being of women survivors of DV living in shelter homes, strengthening their body–mind connection and self-confidence. Based on the principles of psychomotor therapy, FOM uses physical activity, body awareness, and relaxation techniques to help DV survivors safely regain awareness of bodily sensations, integrate these sensations into the sense of body agency, and develop their abilities for self-regulation (19). Each individual or group session sequentially follow three therapeutic steps: warming up; body awareness and grounding; and relaxation.

#### 1.1.1 Warming-up

The initial phase of each session involves activating proprioceptive (muscular) and interoceptive (visceral) sensations through aerobic exercises and strength training, which potentially alleviate PTSD symptoms (22, 25, 26). In FOM’s approach, exercise intensifies neutral bodily sensations to counteract bodily dissociation and hypo-arousal, fostering greater awareness (27). This process is supported by the use of bodily metaphors and movement imagery to deepen body connection and empowerment. Activities are tailored to participants’ abilities and designed to emphasize safety, joy, and process-oriented engagement, reducing dropouts and enhancing motivation (28–30).

#### 1.1.2 Body awareness and grounding

For individuals experiencing dissociative symptoms, fostering sensory awareness in a gradual, integrative, and non-judgmental manner is crucial (4, 24). Postural awareness and grounding techniques often support this by enhancing bodily awareness and strengthening the body–mind connection, contributing to stabilization and a peaceful reconnection with the body (31, 32). In FOM, the therapist guides participants through slow, intentional movements using therapeutic touch (in group, in-person sessions), imagery, or directed focus. For example, prompts such as “Feel the weight of your body against the wall” or questions like “Where in your body do you feel strength/resistance/movement/stillness?” serve as tools to deepen body awareness. These approaches aim to reinforce the mind–body connection, promoting a sense of body ownership and agency (19, 29, 33).

#### 1.1.3 Relaxation

Regulating arousal is a critical focus of interventions for trauma-related disorders (34). Techniques such as relaxation and controlled breathing are commonly used to lower excessive physiological arousal and build emotional regulation skills (35). FOM’s sessions end with relaxation practices rooted in physiological regulation, including progressive muscle relaxation and Wintrebert’s active-passive relaxation (36). Progressive muscle relaxation is introduced early as an accessible, present-focused method that can be adapted for quick, everyday use (37). Once participants become proficient in this technique, the active-passive relaxation method is introduced to deepen relaxation. In the final sessions, participants are encouraged to practice attention regulation exercises to support ongoing arousal regulation in daily life.

In summary, the FOM program offers each woman individual and group sessions, focusing on movement, expression, breathing, and relaxation techniques, with two main goals. The first goal is to gradually foster a non-judgmental awareness of bodily sensations and the connection between sensations and emotions, thereby enhancing the body–mind relationship. The second goal is to improve self-regulation as a means to alleviate mental health symptoms, trauma symptoms and, indirectly, enhance overall quality of life.

### 1.2 Initial feasibility and effectiveness results

FOM has been previously implemented in Portuguese shelter homes, with high acceptability and engagement from the participants, and has proven to be beneficial in improving the health and wellbeing of women survivors of DV (27, 38).

In particular, FOM successfully reduced women’s sedentary behavior, sleep problems, and levels of bodily dissociation, while improving mobility-related quality of life, which are especially important for mental health improvement (27, 38). However, most of these women had children also living in the shelter, who did not participate in any form of therapeutic intervention. As previously mentioned, these children are at a high risk for developmental problems, mental health symptoms and behavioral struggles (10, 13, 14). Therefore, providing the children with a therapeutic intervention as early as possible is a crucial step (13, 15).

Regarding feasibility, FOM had optimal rates of reach and acceptability among the women residing in the shelters. However, some women did not participate due to (i) having just arrived at the shelter when the study began, therefore not being ready for a therapeutic process yet, and (ii) schedule incompatibility. Moreover, 29% of the participants who initiated the program did not complete it, mainly due to relocation (38). To overcome these challenges, it was suggested (38) that future implementations should include videotaped or online sessions to ensure continuity of the intervention upon relocation, and open group sessions to welcome newcomers. Also, in terms of the research method, it was suggested to leverage the 4-week control period, and to cross-culturally adapt FOM to shelter homes in different European countries, given their variability in DV rates, social contexts, and healthcare systems integration (1, 39).

To address those limitations, the authors propose a refined version of FOM–Hybrid-FOM–that includes online sessions to ensure continuity of the therapeutic process upon relocation; open group sessions to welcome newcomers; and groups for children aged 5–8 years, 9–12 and 13–15 years.

### 1.3 Hybrid-FOM

The positive effects and acceptability of FOM on initial small-scale studies (27, 38), support our intention to move forward with improving and extending this psychomotor intervention, attending to the main difficulties identified, while preserving its effective methodological mechanisms and techniques. Therefore, Hybrid-FOM (H-FOM), a hybrid version of Feel-Own-Move that combines online individual therapeutic sessions with open in-person group sessions, provided to women and children living in shelter homes, similarly following the three FOM’s steps. H-FOM is expected to directly inform trauma care system policy, effectively addressing the embodiment and health needs of women and children survivors of domestic violence (DV).

## 2 Study aims

This effectiveness-implementation type I hybrid study design has two simultaneous aims. One is to assess the effectiveness of H-FOM on health and quality of life outcomes of women and children survivors of DV living in shelter homes. The other is to assess the barriers and facilitators for H-FOM widespread implementation and integration of its health and exercise-related mechanisms in trauma care systems.

In specific, the first purpose of the study is to examine if women participants show a decrease in mental health symptoms, somatic complaints, quality of life concerns, sedentary behavior and disconnection from the body, and if children participants show improved social–emotional abilities, wellbeing and physical activity levels, and decreased somatic complaints.

The second purpose is to evaluate participants’ acceptance and engagement with the program during recruitment, implementation and follow-up periods. In parallel, the implementation success according to the shelter professionals and the therapists will be assessed, following the conceptual model of Proctor and colleagues (40) for implementation research in mental health, and RE-AIM recommendations and framework (41).

## 3 Methodology

### 3.1 Study design

**Figure 1:**
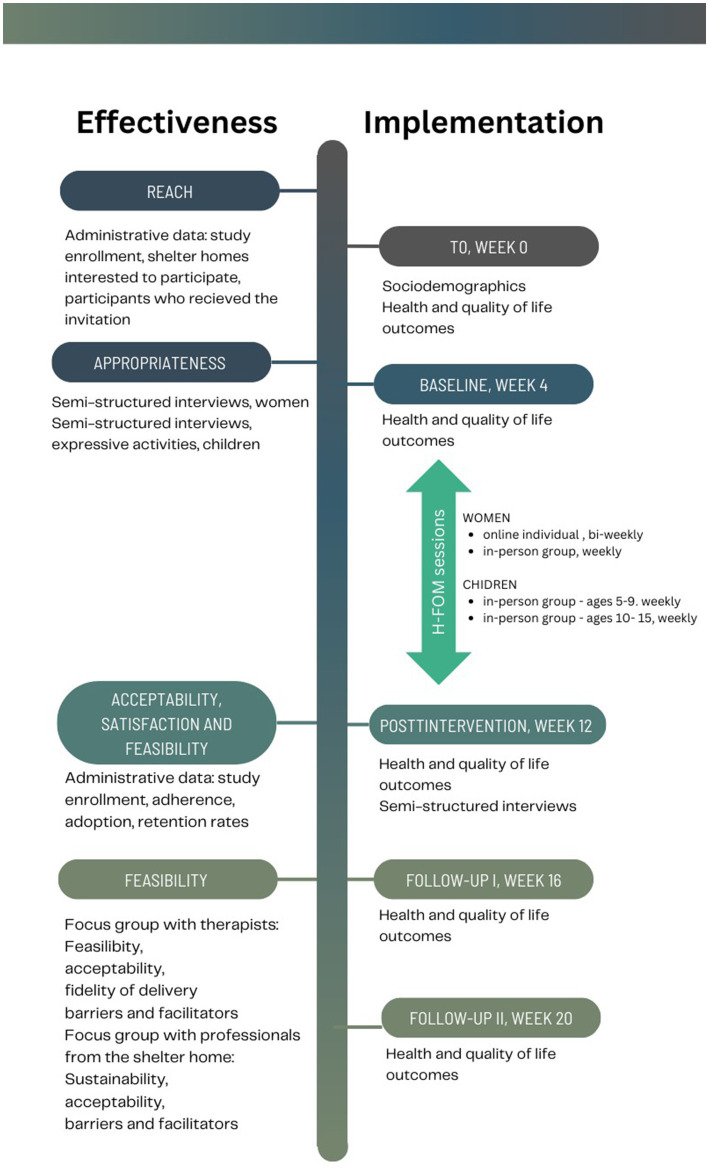
Timeline of the study and effectiveness-implementation assessments.

The current effectiveness-implementation type I hybrid study aims to test the effects of H-FOM on health and quality of life outcomes of women and children while also gathering information on barriers and facilitators for its implementation. Figure 1 schematizes the timeline of the study, including effectiveness and implementation assessment procedures.

### 3.2 Sample and recruitment

The H-FOM study is planned in shelter homes in three Western European countries (Portugal, Spain and the Netherlands), taking advantage of a previously established consortium of trauma-focused research teams with expertise in interventions for trauma. Each research team will contact two shelter homes’ managing entities, inviting them to participate in the H-FOM effectiveness-implementation study. The study characteristics (assessments, activities, place, duration and frequency of the sessions) will be disseminated within the shelter home by managing entities and the researcher. The study aims to recruit a total 100 women and 50 children. Inclusion criteria are having been a victim of domestic violence and being more than 18 years old for women, and between 5 and 15 years old for children and adolescents. Considering previous studies, between 64 and 75% of the participants recruited are expected to complete the program (38, 42). Moreover, at least two professionals from each shelter will be invited to accompany the program and participate in the evaluation of its implementation process.

### 3.3 Procedure

Upon dissemination of the study in each shelter home, women interested in participating, either with or without their children, will sign an informed consent with detailed information about the assessments, activities of the sessions, conditions displayed for the online sessions, and regularity and confidentiality of all the procedures. Following, the initial assessments of sociodemographic and health outcomes will be scheduled with each participant.

After a 4-week control period, the assessments will be repeated prior to the beginning of the intervention, representing the baseline results. The H-FOM will include 8 in-person group sessions for children, 8 in-person weekly group sessions for women, and 16 online individual sessions for each woman. Post-intervention assessments will take place immediately after the intervention, and follow-up assessments at 4 weeks after the intervention. Questionnaires will be filled out online and behavioral measures (namely interoceptive accuracy and physical activity levels) will be assessed in-person, inside the shelter facilities. For the online sessions, shelter homes will be equipped with enough portable devices (tablets) and internet coverage to allow the scheduled sessions of each participant. From the beginning, a safe and private email account will be created for each woman to allow continuity with the online sessions, in case the participant need to be relocated in a different shelter.

After the intervention period, a website with mind–body and physical activity-related resources will be made available for participants, including health-related recommendations, and a portfolio of exercises, accompanied by representative images, videos, and audio recordings for guiding some of the activities.

After the follow-up assessments, focus groups with the participants will be carried out to inform about the barriers and facilitators related to the program and of the use of resources upwards. Recommendations for implementation success will be generated based upon those results.

### 3.4 H-FOM

The traumatic impact of DV often results in sustained neurophysiological hyperarousal or hypoarousal and altered defensive states (4, 21, 34). These altered defensive states require health-related interventions to be facilitated by trauma-informed professionals. To ensure meeting this critical requirement, the researchers and therapists who will implement H-FOM possess the requisite experience and background in mind–body practices for individuals with trauma-related disorders.

#### 3.4.1 H-FOM for women

As previously detailed, H-FOM will combine open group sessions (that allow for new participants in any session), with online individual sessions for the women, which will be adapted to their updated, individual schedules. Thus, H-FOM will expand the possibilities of women with different schedules and shelter stay periods to participate. Each session has three sequential moments: warming-up, body awareness and grounding, and relaxation.

#### 3.4.2 H-FOM for children

H-FOM provides in-person group sessions for children, with the main aim of supporting them in resolving traumatic experiences and social–emotional challenges, through movement and play, which are a child’s primary way of resolving internal conflicts and surpassing difficulties (43, 44). Importantly, each shelter will have the possibility of sampling three groups: one for children aged 5–8 years old, one for children aged 9–12 years old, and another for adolescents aged 13–15 years old. Group sessions will take place in the largest room of the shelter, thereby providing enough safety and privacy conditions for the movement and expressive activities. The children’s sessions, designed to support the resolution of traumatic processes and enhance self-regulation, will follow three phases similar to those detailed above: warming-up and getting in relation, body awareness and self-regulation, and relaxation.

#### 3.4.3 Integrative session

After completion of the program, the dyads (women and their children) who participated will be invited to join a final group session together, which will have the aim of connecting both with their individual processes of finding joy, ease and playfulness on movement, self-regulation and mother–child connection.

### 3.5 Assessments—effectiveness

The effectiveness study follows a non-random within-group repeated measures design. Due to the heterogeneity of the shelter home residents, this study will examine outcomes using a control period for each individual participant, instead of a control group (45). To monitor the control period, participants will be tested at time zero (T0, week 1) and baseline (week 5). Participants will repeat the assessments after they have completed the 8-weekly group sessions and the 16 individual sessions (post-intervention, week 12); then after the first follow-up period (week 16) and after the second follow-up period (week 20).

Sociodemographic data and violence characteristics will be collected to describe the samples of women and children. Health-related outcomes (such as somatic symptoms, post-traumatic stress disorder, anxiety, depression and physical activity levels), embodiment-related outcomes (including interoceptive abilities, body awareness and body dissociation) and quality of life measures will be evaluated to assess the effectiveness of the H-FOM intervention on women. Similar assessments will be conducted with children, with the addition of instruments to evaluate internalizing and externalizing behaviors as part of a broader social–emotional wellbeing measure. Table 1 shows the domains to be assessed in each moment, and if they regard women and/or children. Table 2 details the assessment instruments and respective psychometric properties for each outcome measure. After the intervention, semi-structured interviews with the women and children (separately) will allow for a qualitative analysis of H-FOM effects.

**Table 1:** Sample characteristics and outcome measures of the effectiveness study.

Moment	T0	Baseline	Post-intervention	Follow-up I	Follow-up II
Week	0	4	12	16	20
Sociodemographic data	X				X
Characteristics of violence^a,b^	X	X			X
PTSD symptoms^a^	X	X	X	X	X
Anxiety symptoms^a^	X	X	X	X	X
Depression symptoms^a^	X	X	X	X	X
Somatic complaints^a, b^	X	X	X	X	X
Interoceptive Abilities^a, b^	X	X	X	X	X
Physical Activity levels^a, b^	X	X	X	X	X
Bodily Dissociation^a^	X	X	X	X	X
Quality of Life^a, b^	X	X	X	X	X
Socio-emotional wellbeing^b^	X	X	X	X	X

^aWomen; bChildren.^

**Table 2:** Psychometric properties of the effectiveness assessment instruments.

	Instrument	Cronbach’s alpha		
		Portuguese	Dutch	Spanish
PTSD symptoms				
Women	C-PTSD	0.94	0.79–0.89	0.69–0.87
Children	CTQ-sf	0.66–0.92	0.87	0.66–0.94
Anxiety symptoms				
Women	HADS	0.76	0.78	0.84
Depression symptoms				
Women	HADS	0.81	0.83	0.85
Somatic complaints				
Women	PHQ-15	0.88	0.86	0.84
Children	SCL	0.81	0.84	0.80
Interoceptive abilities				
Women	MAIA	0.61–0.87	0.67–0.89	0.90
Children aged 5–6	JJP			
Children aged >7	MAIA-Y			
Bodily dissociation				
Women	SBC	0.73	0.81	0.62
Quality of Life				
Women	WHOQoL	0.64–0.87	0.66–0.80	0.75–0.80
Socio-emotional wellbeing				
Children	CBCL	0.61–0.83	0.69–0.88	0.71–0.75

C-PTSD, post-traumatic stress disorder checklist–civilian version (51–53); CTQ, childhood trauma questionnaire (54–56); CBCL, child behavior checklist (57–59); HADS, hospital anxiety and depression scale (60–62); JJP, the adapted Jumping Jack Paradigm (63); MAIA, multidimensional assessment of interoceptive awareness (64–66); PHQ-15, patients health questionnaire (67–69); SBC, scale of body connection (70–72); SCL, somatic complaints list (73–75); WHOQoL, World Health Organization Quality of Life questionnaire (76–78).

### 3.6 Assessments—implementation

A mixed methods approach will be used to examine the characteristics, barriers and facilitators of H-FOM implementation within the shelter home context, including professionals, participants and psychomotor therapists. Following the implementation science model of Proctor and colleagues (40), and the Reach, Effectiveness, Adoption, Implementation and Maintenance (RE-AIM) framework (41), will allow for a systematic evaluation of the implementation outcomes, thereby contributing to a discussion about H-FOM scale-up sustainability. The recommended outcome measures of implementation are detailed in Table 3.

**Table 3:** Outcome measures of the implementation study.

Moment	T0		Post-intervention	Follow-up II
Week	0	3	12	20
Reach^a,b^	X	X		
Appropriateness^a,b^		X		
Acceptability^a,b^			X	
Adherence^a^			X	
Retention^a^			X	X
Satisfaction^a^			X	
Feasibility^a,b,c^			X	

^aParticipants; bShelter home professionals; cPsychomotor therapists.^

The appropriateness of the intervention will be assessed through focus groups with the participants during the control period (41). Acceptability, satisfaction, feasibility and reach will be assessed through administrative data (study enrollment, adherence and attendance at individual and group sessions) and participants’ self-report measures (feasibility and satisfaction survey). Sustainability will be evaluated after the follow-up period using focus groups with the professionals from the shelters. Additionally, focus groups with the psychomotor therapists will explore their perceptions regarding H-FOM feasibility, acceptability, fidelity of delivery, and barriers and facilitators of the interventions. All focus groups will be based on simple semi-structured interviews, and they will be audio-recorded, transcribed verbatim, and anonymized.

### 3.7 Sample size and power

WebPower was used to calculate the minimum required sample size for a repeated-measures study. For this calculation, significance level (alpha) was set at 0.05, power at 90%, with 1 group, 4 measurements, and a within effect. A minimum of 58 participants is required.

### 3.8 Data handling and analysis plan

#### 3.8.1 Effectiveness

A descriptive analysis of sociodemographic and health variables will be performed. The normality of data will be checked through the Shapiro–Wilk test. Missing values should represent less than 5% of the data, and Little’s MCAR test must have *p* > 0.05, indicating that these are missing at random. If so, missing values will be replaced by the mean value of the respective item scores. All statistical analyses will be conducted using version 28.0 of SPSS and significance level will be set at *p* < 0.05.

A one-way repeated measures ANOVA will be used to examine within-group changes between the different moments (T0, Baseline, Post-intervention, follow-up I and follow-up II). The Bonferroni correction will be used to adjust significance levels, considering significance if *p* < 0.05.

Effect sizes will be provided as partial eta-squared (*η_p_^2^*) and interpreted as: 0.01–0.06, small effect, 0.06–0.14, medium effect, and ≥ 0.14, large effect (46). Results of non-parametric variables will be presented as median and interquartile range (IQR). Friedman tests will be carried out to examine changes in non-parametric variables, using *post hoc* pairwise comparisons (Wilcoxon Signed-Rank test) and a Bonferroni adjustment with significance levels considered at *p* < 0.017. Effect sizes will be calculated using Kendall’s W Value, and interpreted as <0.3, small effect, 0.3–0.5, moderate effect, and >0.5, large effect (47). The delta value (*Δ*%) of proportional change between each moment will be calculated using the formula:


$$\Delta \%=\left[\left(\mathrm{momentY}–\left(\mathrm{momentY}-1\right)\right)/\left(\mathrm{momentY}-1\right)\right]\times 100.$$

#### 3.8.2 Implementation

Focus group audio recordings will be transcribed verbatim. The *corpus* will be analyzed using a deductive (theory-driven) content analysis, guided by the study’s aims of identifying implementation characteristics, barriers and facilitators. Analyses will be carried out independently by two researchers, and a third researcher will resolve disagreements. A mixed-methods approach will be employed to integrate findings on both effectiveness and implementation. The design follows a sequential structure (QUAN→ qual) where qualitative data collected from participants, therapists and shelter home personnel will be used to contextualize and interpret the quantitative results from the feasibility and effectiveness studies (48, 49). Moreover, semi-structured interviews with the women and children (separately) will allow for a qualitative analysis of H-FOM effects.

## 4 Discussion

Considering DV as a worldwide problem with a broad impact on health and wellbeing of women and children, it has been recommended that the support for victims of DV should encompass a more holistic approach to their health, including physical activity, body awareness, expressive movement, and relaxation (4, 21, 22, 30, 50). These dimensions are integrated in the FOM approach, which has proven effective in improving the health and quality of life of women living in shelter homes. However, relying solely on in-person sessions has an associated risk of disruption of the process when women are relocated. H-FOM aims to address this problem, by including individual online sessions, and open in-person sessions that allow for newcomers in the shelter, thereby providing a facilitating strategy to engage and ensure continuity and success of the therapeutic process.

Moreover, by adding an intervention targeting children, H-FOM will support these children to transform the meaning of the shelter stay, develop healthy relationships with their peers, and resolve internal conflicts, often neglected by the fact of them being considered indirect victims.

This psychomotor therapy approach, through its specific aims and mechanisms, has proven effective in reducing levels of bodily dissociation, which is of paramount importance in the field of DV. It is particularly relevant to the public health and social goal of breaking the cycle of violence. Recent studies have highlighted dissociation as a significant mediator in the revictimization of women who were abused during childhood (17). In fact, dissociation often manifests in adolescents as a consequence of childhood traumatic experiences and serves as a risk factor for becoming victim of intimate partner violence in adulthood. Therefore, a psychomotor intervention that reduces bodily dissociation holds promise in breaking the cycle of violence. If implemented at earlier developmental stages, preferably immediately after the first traumatic experiences, H-FOM could be a promising strategy in health and social care.

No study is without challenges and limitations. Specifically, the online component of H-FOM requires shelters to be equipped with electronic devices and stable internet access while ensuring privacy, confidentiality and online security, which entails financial costs and significant digital safety measures. For the therapeutic group sessions for children, the main challenge will be securing a private space and dedicated time within the shelter, allowing children to freely explore different movement modalities and express their emotions. Finally, the study’s use of a control period and a repeated measures design with follow-up poses the risk of a higher drop-out rate due to the many assessment moments. This risk can be mitigated by using shorter versions of each scale or instrument.

This study will therefore contribute to trauma support services and associated healthcare responses to address the need for a more physically active and body-centered approach.
