# Correction: Hybrid Feel-Own-Move^®^: protocol for an effectiveness-implementation study of a psychomotor intervention for survivors of domestic violence

**DOI:** 10.3389/fpubh.2025.1713973

**Published:** 2025-10-27

**Authors:** Joana Machorrinho, Guida Veiga, José Marmeleira, Mia Scheffers, Graça Duarte Santos

**Affiliations:** ^1^Comprehensive Health Research Center, Universidade de Évora, Évora, Portugal; ^2^Departamento de Desporto e Saúde, Escola de Saúde e Desenvolvimento Humano, Universidade de Évora, Évora, Portugal; ^3^School of Human Movement and Education, Windesheim University of Applied Sciences, Zwolle, Netherlands

**Keywords:** body–mind intervention, therapy, intimate-partner violence, women, children, hybrid, healthcare, shelter homes

An incorrect **Funding** statement was provided. The previous **Funding** statement read:

“The author(s) declare that financial support was received for the research, authorship, and/or publication of this article. This research was funded by Fundação para a Ciência e Tecnologia, IP National support through Comprehensive Health Research Centre, CHRC (UI/BD/150985/2021). Also, this publication is funded by national funds through the Foundation for Science and Technology, under the project UIDP/04923/2020.”

The correct funding statement reads:

The author(s) declare that financial support was received for the research, authorship, and/or publication of this article. This research was funded by Fundação para a Ciência e Tecnologia, IP National support through Comprehensive Health Research Centre, CHRC (UI/BD/150985/2021). Also, this publication is funded by national funds through the FCT–Fundação para a Ciência e Tecnologia, I. P., in the framework of the CHRC UID/04923/2025.

The original version of this article has been updated.

